# Cancer services in Sri Lanka: current status and future directions

**DOI:** 10.1186/s43046-021-00070-8

**Published:** 2021-06-03

**Authors:** Umesh Jayarajah, Anuruddha M. Abeygunasekera

**Affiliations:** Department of Urology, Colombo South Teaching Hospital, Kalubowila, Western Province Sri Lanka

**Keywords:** Cancer, Sri Lanka, Cancer care, Cancer burden

## Abstract

**Background:**

The burden of cancer in Sri Lanka is on the rise. The overall incidence of cancer in Sri Lanka has doubled over the past 25 years with a parallel rise in cancer-related mortality. Cancer has become the second commonest cause of hospital mortality in Sri Lanka. In this review, we aim to provide an overview of the current status and future direction of cancer care in Sri Lanka.

**Main body:**

In Sri Lanka, cancer services are predominantly provided by the state sector free of charge to the general public. With the establishment of national cancer policy on cancer prevention and control, there has been a commendable improvement in the cancer services provided island-wide. An increasing number of breast, oropharyngeal, thyroid, oesophageal, colorectal, lung, and gastric cancers are being diagnosed and treated annually. Primary prevention measures include restrictions in tobacco and HPV vaccination. Screening programs for selected cancers such as breast, oral and cervical cancers are delivered. Medical oncology units with facilities for systemic therapy and adequately supported by surgical, pathology, and radiology departments have been established in each district general hospital island-wide. Although the current progress is commendable, future changes are necessary to overcome the current limitations and to cater the ever increasing burden of cancer. Measures are necessary to enhance the coverage of Sri Lanka Cancer Registry. Timely high-quality research and audits are essential. Community participation in planning strategies for cancer prevention and treatment is minimal. Community-based palliative care facilities and radiation and other systemic therapy should be made available in all provinces. A culture of multi-disciplinary care with proper referral pathways would help to improve the current setting.

**Conclusion:**

In conclusion, Sri Lanka has a reasonably balanced and continuously expanding program for prevention, screening, and treatment of cancers. Emphasis on preventive strategies related to reducing tobacco smoking, chewing betel, and obesity, making cancers a notifiable disease, involving the community in planning cancer care and prevention strategies, conducting research to evaluate cost-effectiveness of existing treatment and increasing radiotherapy facilities would further improve the cancer services in Sri Lanka.

## Background

Asia is the largest continent in the world with countries showing a wide diversity of sociocultural behavior, economic performance, and health-care systems. Sri Lanka is an island nation in South Asia, a developing country with a lower-middle-income economy and a population of 22 million. The Sri Lankan government comprises of a parliamentary democratic system led by an executive president and a cabinet of ministers. Sri Lanka is divided into 9 provinces administered by provincial councils and 25 districts.

Sri Lanka has a pluralistic healthcare system with the majority (95%) of the patients seeking treatment through allopathic healthcare providers complimented by the Ayurvedic and complementary medicine [1]. Sri Lankan state sector offers free universal healthcare to all citizens and is responsible for 90–95% of inpatient care and preventive health at community level [1]. State sector healthcare workers are employed full time by the Ministry of Health with a monthly salary. However, they are allowed to engage in private healthcare provision outside working hours according to a fee-for-service model and only a minority of expenses are covered by private health insurance [2]. Private sector healthcare institutes are confined to major cities of the country and focus mainly on curative services. The allopathic free healthcare system governed by the state sector is mainly comprised of preventive and curative sectors [1]. The curative health sector is comprised of primary secondary and tertiary care hospitals situated island-wide [1]. Preventive and promotive health at community level is governed by more than 350 Medical Officers of Health (MOH) Units. Total health expenditure as a share of gross domestic product (GDP) in Sri Lanka is comparatively low, approximately 4% compared to 6% in Egypt, 17% in USA, and 3.6% in neighboring India [3, 4]. With universal healthcare and a robust public health network across the country, Sri Lanka has made noteworthy achievements in health outcomes compared to other developing countries despite a low total health expenditure [5]. Sri Lanka is known for its high life expectancy, low maternal and child mortality, and decreasing levels of communicable diseases. The average life expectancy is 75 years for Sri Lankans while in neighoring India, it is 70.8 years and in Egypt it is 72 years [3, 4]. The infant mortality rate in Sri Lanka is 8.4 while it is 19 in Egypt and 39.1 per 1000 live births in neighbouring India [4].

While this success is laudable, the country needs to strengthen the existing health system to face changes brought by demographic and epidemiological transitions.

**Fig. 1:**
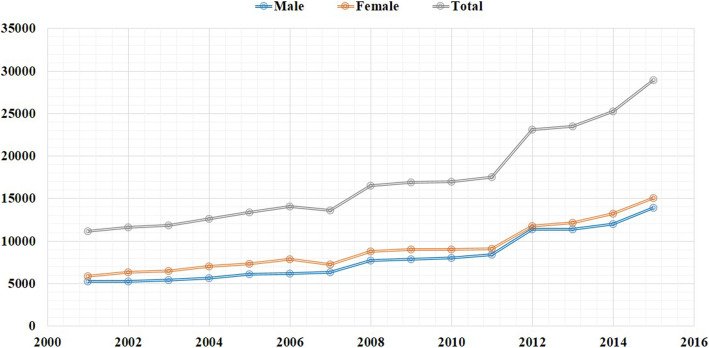
The total number of cancers detected from 2001 to 2015

Cancer burden is a measure of the incidence of cancer within the population and an estimate of the financial, emotional, or social impact it creates [4]. According to the WHO approximately 70% of deaths due to cancer occur in low and middle-income countries [4]. Increasing burden of cancer has become a major challenge faced by Sri Lanka (Fig. 1). The overall incidence of cancer in Sri Lanka has doubled over the past 25 years with a parallel rise in cancer-related mortality [2]. Cancer has become the second commonest cause of hospital mortality in Sri Lanka by constituting 14% of all hospital deaths [2]. According to GLOBOCON estimates for Sri Lanka, 23,530 new cases and 14,013 deaths would have occurred due to cancer in year 2018 and this is expected to increase by 23% every year till 2030 [6, 7]. Breast cancer is the commonest cancer and the incidence is rising [8]. Other common cancers include oropharyngeal, thyroid, oesophageal, colorectal, laryngeal, lung, and gastric cancers, which also show an increasing incidence [9–16]. This may partly reflect a rise in true incidence. However, the efforts of the National Cancer Control Programme (NCCP) in increasing facilities for better diagnosis and reporting may have contributed to the current trend. We aim to discuss the current status and future direction of cancer care in Sri Lanka as clinicians who deal with cancer patients.

## Main text

### Current status of cancer services in Sri Lanka

With the establishment of a national cancer policy on cancer prevention and control, there has been a commendable improvement in the cancer services provided [2, 17]. In 1980, NCCP was established as the central institution for prevention and control of cancers. The island-wide cancer database known as Sri Lanka Cancer Registry is under the responsibility of the NCCP. Evidence-based strategies have been implemented for prevention and to increase the awareness of cancer among the public and primary healthcare personnel [2].

### Primary prevention

Tobacco use, alcohol, betel quid chewing, and obesity due to physical inactivity and unhealthy diet are the main preventable causes of cancer in Sri Lanka [6, 18]. Role of insecticides, pesticides, and food additives in causation of cancer is debatable [6, 19]. Betel quid chewing and selling of betel quid, tobacco, and areca nut products were banned in healthcare facilities in 2018 and in all government institutes in 2019 [6]. Tobacco smoking is banned in public places in Sri Lanka. Advertising related to tobacco products is prohibited in all forms of public media. Although smokeless tobacco and betel chewing are major predisposing factors for common cancers in Sri Lanka, due to cultural beliefs and habits, there are practical issues in banning them completely. Betel is considered a sign of prosperity and good luck in Sri Lanka. National immunization program in Sri Lanka started HPV vaccination in 2017 to 10–11-year-old girls as a school-based vaccination program. This is expected to reduce the number of cervical cancers in the future which contributes to 5% of all cancers now [20]. Vaccination against hepatitis B has been incorporated into the national vaccination schedule for many years and has achieved a target of 99% [4].

Although there is considerable emphasis on reduction of exposure to carcinogens, greater emphasis is needed to control metabolic risk factors such as obesity, physical inactivity, and unhealthy diet which are major modifiable risk factors for commonest cancers in Sri Lanka such as breast and gastrointestinal cancers. Educating the general public via mass media and social media and encouraging a healthy life style is necessary for prevention of common cancers.

### Screening

Screening programs for selected cancers such as breast, oral, and cervical cancers are delivered by the NCCP [2]. Recently, NCCP published guidelines for early detection and referral of seven common cancers in Sri Lanka [6]. Although breast cancer is the commonest cancer in Sri Lanka, still the country does not have enough machines and personnel to induct a nationwide program of mammographic screening. As an alternative breast self-examination is promoted while mammographic screening is done in 18 state hospitals and few centers in the private sector hospitals [5, 6]. Furthermore, clinical breast examination is freely offered in community-based clinics for women; however, such facilities are underutilized. Similarly, screening facilities for oral cancers are available in community-based health centers nationwide and the utility seems to be suboptimal [21]. Future studies are needed to quantify the usage of such resources and to identify measures needed for improvement. Pap smear screening is done to identify cervical carcinoma early through community-based clinics for women located throughout the country. In 2016, there were 850 such well women clinics and cohorts of women aged 35 and 45 years are the two target groups for screening [6].

Although prostate cancer is the fifth common cancer among men in Sri Lanka there is no nationally accepted screening program using serum PSA. However, facilities to test serum PSA level are available widely across the country in both government as well as private laboratories making opportunistic screening of prostate cancer possible. This facility was made available recently and may help to increase the number of prostate cancers diagnosed at an early stage. At present, about 60% of prostate cancers are diagnosed at the metastatic stage [22].

Endoscopic screening is established in developed countries with high prevalence of gastrointestinal malignancies [23]. Currently, endoscopic screening programs for gastrointestinal malignancies for upper and lower gastrointestinal tumors are not established in Sri Lanka due to constraints in cost and resources. However, due to increasing incidence of oesophageal, gastric, and colorectal cancers, endoscopic screening programs may need to be considered in the future [12, 13, 15].

### Diagnosis and treatment

National Health Service of Sri Lanka which provides healthcare to the vast majority of people is free at the point of delivery. Although private sector hospitals which provide services to the more affluent are available throughout the country, specialized inpatient cancer services are limited to a few major cities and only a minority of patients are able to access systemic therapy in the private sector due to high cost [2]. Nevertheless, private sector healthcare facilities are available for outpatient cancer services and surgical treatment across the country. Although waiting times for treatment are negligible in the private sector, there are no publications related to outcome of patients treated in private hospitals making it impossible to make valid comparisons.

**Fig. 2:**
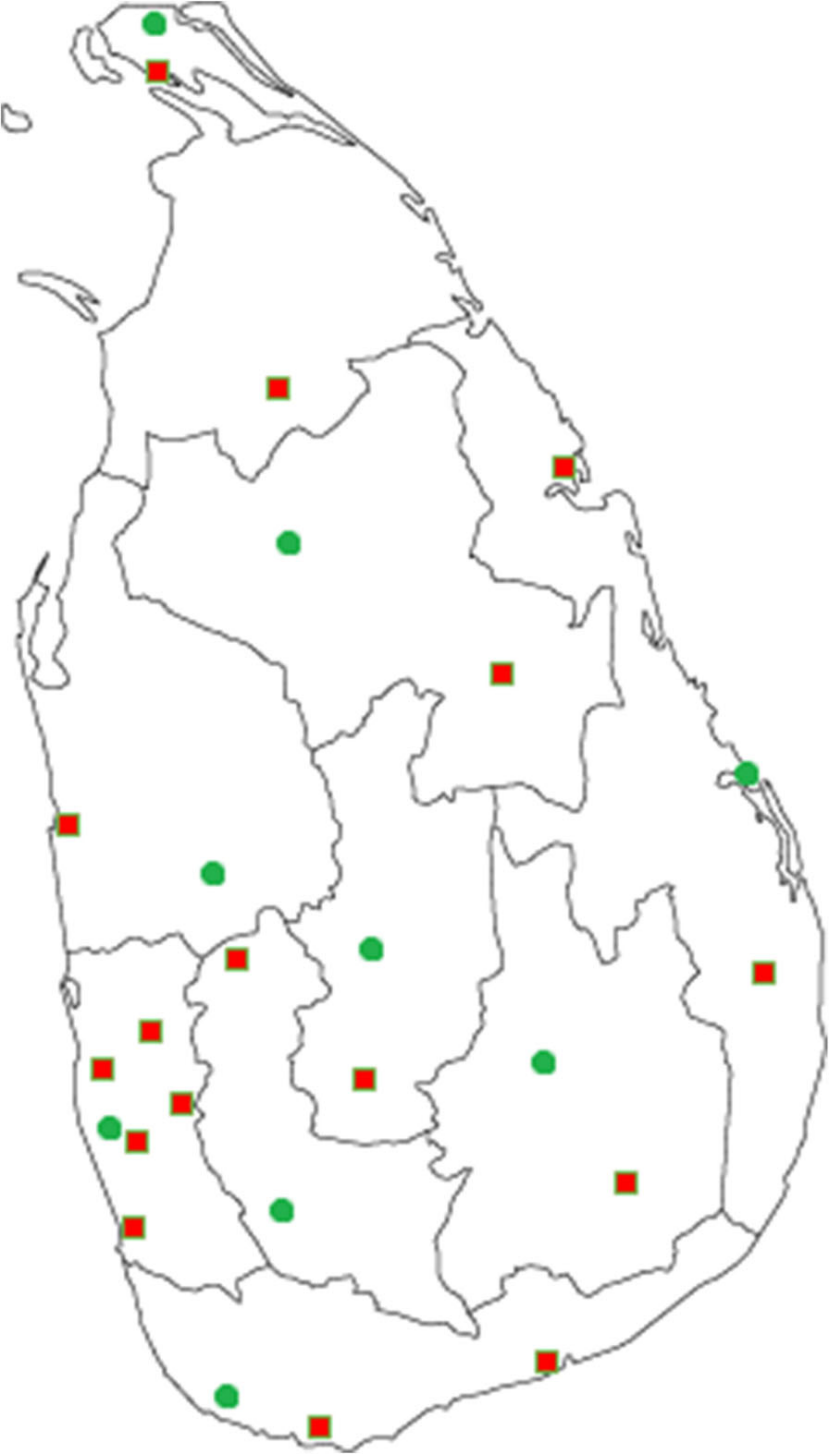
Distribution of main cancer treatment centers (green circle) one in each province and district treatment centers (red square) in Sri Lanka

Facilities at tertiary care hospitals have been improved to manage all cancers in an evidence-based manner and to improve overall quality of cancer care (Fig. 2). Medical oncology units with facilities for systemic therapy have been established in each district general hospital island-wide [24]. Even the latest chemotherapeutic agents like tyrosine kinase inhibitors and check point inhibitors are available in the country. Furthermore, specialized surgical oncology units have been established in nine provincial hospitals to treat cancer patients [24]. However, radiation oncological facilities are available only in seven provincial hospitals. Currently, four linear accelerators are available and steps are underway to procure more machines and to establish a radiation oncology center in each province [24]. Only two private sector hospitals in the capital city of Colombo have facilities for radiation therapy. Sri Lanka requires at least one radiotherapy machine per million population which will require 10–12 machines more [2, 17]. Limitation of radiation therapy to a few selected hospitals has led to long waiting times for patients requiring such services [2, 17]. Patients from rural areas are forced to travel long distances which may lead to a higher default rate. The recent attempt to provide radiation oncological facilities to all nine provinces is a welcome move [2, 17].

With the enhancement of facilities for minimally invasive radiologically guided tissue sampling methods, the diagnosis and reporting of these malignancies could be further improved. Immunohistochemistry facilities, although not widely available, have helped precise histopathological diagnosis of complex malignancies which is essential for selection of appropriate therapy [25]. However, facilities for molecular diagnostics which is considered vital for precision therapy of malignancies is severely restricted in state hospitals and limited to basic facilities available at Medical Research Institute, Sri Lanka [26]. Health ministry should consider establishing few such centers in premier state hospitals a priority.

Multi-disciplinary cancer care is less commonly practiced in Sri Lanka [6]. Various specialities function independently when managing the same patient. This is especially seen when patients are referred to a different unit to treat the same condition. Ideally, a formal agreement in the treatment plan should be reached after the input from all relevant specialities across different centers [27]. Formal pathways should be established for referrals and proper documentation should be given to the patients once treatment is completed and referred back [6]. Although breast and thyroid are commonest cancers in Sri Lanka, there are no dedicated breast and endocrine cancer centers. Furthermore, there are no centralized and dedicated cancer centers for urological malignancies manned by urological surgeons specialized in uro-oncology [28]. Lack of community-based nursing care service essential for stoma management, tracheostomy care, and wound care is an unmet need.

Majority of cancers are treated based on globally accepted guidelines formulated by well-recognized authorities from the Western world [29, 30]. Some of the newer therapeutic modalities related to precision therapy and targeted therapy are not available in Sri Lanka due to cost constraints and the limited benefits in terms of improvement of outcomes. Already, brief local guidelines have been developed covering certain aspects of management of common cancers including breast, oral, cervical, esophageal, colorectal, thyroid, and prostatic carcinoma by the NCCP [6]. Much more comprehensive guidelines covering all important aspects of patient care should be prepared by an established authority, considering the locally available resources similar to these documents.

Treatments with minimal benefit can cause disproportionately more hardships to patients and their carers affecting the quality of life as well as becoming a financial burden [27]. As a country with a developing economy, we should always try to adopt cost-effective alternatives despite western guidelines and pressure from big pharma. For example, despite scientific evidence in favor of surgical orchiectomy as a means of androgen deprivation therapy for metastatic prostate carcinoma, still much more expensive gonadotrophin-releasing hormone (GnRH) agonists are used in Sri Lanka [31].

In reality, most cancer patients seek services of alternative medical practitioners [17, 32]. Certain native/Ayurvedic treatments may help palliate their symptoms especially the undesirable adverse effects of chemotherapy [33]. Collaboration with institutes of indigenous medicine to harness such positive aspects may also give an opportunity to regulate the minority of native healers who proclaim ineffective cancer cures.

### Cancer registry and research

Since its inception in 1985, the National Cancer Registry maintained by the NCCP is the most comprehensive cancer database in Sri Lanka. To date, the incidence of all cancers in Sri Lanka from 2001 to 2014 are summarized and published online. Population-based data on incidence and mortality is available for the Colombo District [6]. It is estimated that in 2014, a national coverage of 80% was achieved and present coverage is likely to be more [2].

Despite the rapid expansion of the Sri Lanka Cancer Registry, certain cancers such as prostate and non-invasive bladder cancers are under-reported [19, 22, 34, 35]. This is mainly because these early cancers are usually treated solely by the clinicians without referring to the cancer centers. Cancer registries are being established at all tertiary care hospitals and data is gathered from histopathology laboratories to overcome this limitation. Low incidence of hepatobiliary cancers compared to the neighboring countries may be due to the lack of histological diagnosis and reporting, precluding inclusion to the cancer database [36, 37]. Collection of data regarding patients treated in the private sector institutes is difficult. This may be overcome by making cancer a notifiable disease so that reporting will be mandatory even in the private sector hospitals. Recently, NCCP has initiated an electronic data gathering system using mobile applications to enhance reporting of vital data at all levels. This type of user-friendly technology has already been shown to be cheap and effective for collection of cancer data [38]. Measures should be taken to expand the electronic data gathering system to all dedicated oncology centers and to clinicians who manage these patients.

Timely research and audit programs are lacking. Research on cancer care and quality improvement are quite feasible in a country like Sri Lanka, as a majority of patients are treated and followed up in a few specialized centers limited to a smaller geographical area. Timely audit of cancer epidemiology and treatment outcomes is essential to guide primary prevention and screening programs, allocation of resources, and revisions of guidelines. Characteristics of malignancies of this part of the world could be different to the published data from the developed world [18, 39–41]. One such prominent example is extreme rarity of carcinoma-in-situ of bladder in Sri Lanka which has been attributed to widespread BCG vaccination at birth [38, 42]. Although the Ministry of Health allocates nearly 2.8% of its total recurrent expenditure budget to the premier cancer hospital in the country, there is a paucity of published data to prognosticate the outcome of diseases managed at National Cancer Institute Sri Lanka (NCI), presently known as Apeksha Hospital. Furthermore, studies on quality of life and psychological impact of common cancers are also lacking [43, 44]. Hopefully, recently established research unit of the NCI would fill this void.

### Health literacy and public education

The improvement of health literacy and public education programs appear to have reduced the default rate and social stigma due to cancer. It is equally important to remember health education related to cancers and cancer screening should be done by experts who are skilled in providing the appropriate mixture of information without inducing unnecessary and harmful anxiety among the healthy members of the society [45]. Otherwise, psychological morbidity and its consequences on the masses may surpass the benefits of early detection of few cancers. There is a lack of good quality online information for cancer patients [46]. Studies have shown that the currently available online information on cancer is unreliable and of poor quality and readability [47, 48]. Although, the NCCP has initiated to publish online leaflets for common cancers, more comprehensive material on common cancers designed by local experts describing the available treatment, side effects, and outcomes will be useful. Involving the communities in planning cancer care prevention strategies would be a useful initiative. This would help to demystify treatment and allow online messaging to be developed in terms grounded in community wisdom and terminology. Furthermore, International Agency for Research in Cancer (IARC) would be a useful resource for evidence-based communication that might be adapted, with help from communities.

### Palliative care

A substantial emphasis has been placed on curative services by the authorities in recent years. However, services on palliative care need considerable improvement, although it has already been established in selected tertiary care hospitals. Some patients with end-stage disease are referred back to the local hospital which lack palliative care specialists and resources. A community-based palliative care service with adequate resources would improve the quality of life in these patients. A palliative care team can be attached to every oncology clinic which can coordinate and guide the community-based palliative care service. Narcotic analgesics such as oral morphine, which are currently restricted due to the fear of abuse, could be made available through these dedicated centers.

## Conclusions

In conclusion, Sri Lanka has a reasonably balanced and continuously expanding program for prevention, screening, and treatment of cancers. Although the current progress is commendable, future advances are necessary to overcome existing limitations and to cater the increasing burden of cancer. Special emphasis on preventive strategies related to reducing tobacco smoking, chewing betel quid, and obesity, making cancers a notifiable disease, involving the community in planning cancer care and preventive strategies, conducting research to evaluate cost-effectiveness of existing treatment and increasing the number of radiotherapy machines would further improve the cancer care in Sri Lanka.

## Data Availability

The datasets supporting the conclusions of this article are available from the corresponding author on reasonable request.

## Abbreviations

MOH: Medical Officers of Health
GDP: Gross Domestic Product
NCCP: National Cancer Control Programme
NCI: National Cancer Institute
GnRH: Gonadotrophin-releasing hormone
IARC: International Agency for Research in Cancer
